# Left atrial effective conducting size predicts atrial fibrillation vulnerability in persistent but not paroxysmal atrial fibrillation

**DOI:** 10.1111/jce.13990

**Published:** 2019-06-18

**Authors:** Steven E. Williams, Louisa O’Neill, Caroline H. Roney, Justo Julia, Andreas Metzner, Bruno Reißmann, Rahul K. Mukherjee, Iain Sim, John Whitaker, Matthew Wright, Steven Niederer, Christian Sohns, Mark O’Neill

**Affiliations:** ^1^ Division of Imaging Sciences and Biomedical Engineering King's College London London United Kingdom; ^2^ Department of Cardiology Asklepios Klinik St. Georg Hamburg Germany; ^3^ Clinic for Electrophysiology Herz‐ und Diabeteszentrum NRW, Ruhr‐Universität Bochum Bad Oeynhausen Germany

**Keywords:** atrial fibrillation vulnerability, conduction velocity, left atrial effective conducting size, refractoriness

## Abstract

**Background:**

The multiple wavelets and functional re‐entry hypotheses are mechanistic theories to explain atrial fibrillation (AF). If valid, a chamber's ability to support AF should depend upon the left atrial size, conduction velocity (CV), and refractoriness. Measurement of these parameters could provide a new therapeutic target for AF. We investigated the relationship between left atrial effective conducting size (LA_ECS_), a function of area, CV and refractoriness, and AF vulnerability in patients undergoing AF ablation.

**Methods and Results:**

Activation mapping was performed in patients with paroxysmal (n = 21) and persistent AF (n = 18) undergoing pulmonary vein isolation. Parameters used for calculating LA_ECS_ were: (a) left atrial body area (*A*); (b) effective refractory period (ERP); and (c) total activation time (*T*). Global CV was estimated as √A/T. Effective atrial conducting size was calculated as ${\mathrm{LA}}_{\text{ECS}}=A/\left(\mathrm{CV}\times \mathrm{ERP}\right)$. Post ablation, AF inducibility testing was performed. The critical LA_ECS_ required for multiple wavelet termination was determined from computational modeling. LA_ECS_ was greater in patients with persistent vs paroxysmal AF (4.4 ± 2.0 cm vs 3.2 ± 1.4 cm; *P* = .049). AF was inducible in 14/39 patients. LA_ECS_ was greater in AF‐inducible patients (4.4 ± 1.8 cm vs 3.3 ± 1.7 cm; *P* = .035, respectively). The difference in LA_ECS_ between inducible and noninducible patients was significant in patients with persistent (*P* = .0046) but not paroxysmal AF (*P* = .6359). Computational modeling confirmed that LA_ECS_ > 4 cm was required for continuation of AF.

**Conclusions:**

LA_ECS_ measured post ablation was associated with AF inducibility in patients with persistent, but not paroxysmal AF. These data support a role for this method in electrical substrate assessment in AF patients.

## 1 INTRODUCTION

The multiple wavelet hypothesis, initially proposed by Moe et al^1^ in the 1950s, is an established mechanistic theory to explain the maintenance of atrial fibrillation (AF). According to this theory, sustained fibrillatory activity is the result of multiple random independent wavefronts propagating across an altered electrical substrate which maintain fibrillation after the initial trigger is extinguished. Support for the multiple wavelet hypothesis was originally demonstrated in a canine model of cholinergic AF,^2^ but more recently both electrocardiographic imaging during AF in patients with paroxysmal, persistent and long‐standing persistent AF,^3^ and epicardial mapping in patients with persistent AF undergoing open heart surgery^4^ have further supported this mechanism in human AF.

Another important mechanistic theory of AF, that of “functional re‐entry,” was proposed by Allessie et al^5^ in an ex vivo study of rabbit atria. In this study, rotating re‐entry occurred in the absence of an anatomical obstacle. Subsequent studies further demonstrated functional, anatomic, and micro re‐entry to be driving mechanisms in animal models of AF.^6^, ^7^ In the modern era two new technologies (intracardiac phase mapping^8^ and electrocardiographic imaging^9^) have also been used to demonstrate, albeit controversially, the occurrence of rotors in human AF, described previously as drivers of function re‐entry.^10^

Since directly mapping activation in AF is challenging, our ability to deliver therapies based on such mechanisms is currently limited. Nevertheless, a key concept of these hypotheses is that a critical mass of tissue is required to facilitate the perpetuation of AF.^11^ Whether chamber size determines the capacity of the chamber to sustain multiple re‐entrant wavelets will depend on the electrical properties (conduction velocity [CV] and refractoriness) of that chamber.^12^ A technique to quantify the electrical substrate size of an atrium, based on a limited set of measurements collectible within a clinically applicable timescale, would allow interventional AF treatments to be individualized. We therefore propose a metric termed “Effective Conducting Size” (units, cm) which is a function of total atrial area, CV, and a single‐site measurement of refractoriness. We hypothesize that the ability of a chamber to support AF is related to Effective Conducting Size, independent of the use of antiarrhythmic medication.

## 2 MATERIALS AND METHODS

### 2.1 Patient selection and clinical procedures

Adult patients with AF undergoing first‐time ablation were eligible for inclusion. The research protocol conformed to principles outlined in the Declaration of Helsinki and ethical approval was granted by the National Research Ethics Service. Written informed consent was obtained from all participants before the study. Patients with prior atrial ablation were excluded. Following femoral venous access, a decapolar (St. Jude Medical, St. Paul, Minnesota) catheter was positioned in the coronary sinus. Left atrial access was obtained via a trans‐femoral approach following a trans‐septal puncture. A circular mapping catheter (Lasso; Biosense Webster, Diamond Bar, CA) and an 8Fr ablation catheter (Thermocool, SmartTouch; Biosense Webster) were advanced into the left atrium via two 8.5Fr SR0 sheaths. All patients were anticoagulated intraprocedurally targeting an activated clotting time of >300 milliseconds.

### 2.2 Left atrial mapping and pacing protocol

Before ablation, all patients underwent left atrial activation and voltage mapping using the Carto electro‐anatomic mapping system (Biosense Webster, Diamond Bar, CA) during coronary sinus pacing at a cycle length of 600 milliseconds. For patients who presented in AF, direct current cardioversion was performed. Wide‐area encirclement of the pulmonary veins was performed in power‐controlled mode using a contact force‐sensing ablation catheter. Entrance and exit block were demonstrated to confirm pulmonary vein isolation and additional ablation performed in the case of reconnection. Following successful pulmonary vein isolation, the ablation catheter was positioned in the center of the posterior left atrial wall. Once adequate contact force (>3 g) and catheter stability were achieved, a pacing protocol was applied consisting of an 8‐beat drive train at a basic cycle length (BCL) of 600 milliseconds and an extra stimulus with coupling intervals reducing from 450 milliseconds in 10 millisecond steps. The atrial effective refractory period (ERP) was defined as the longest extra stimulus coupling interval failing to capture the atrium.

### 2.3 AF vulnerability

Following ERP measurements, an AF induction protocol was performed by pacing from the ablation catheter at the left atrial posterior wall. The protocol consisted of sensed doubles (S1 = 600 milliseconds; S2 = 400 milliseconds, decreasing in 10 milliseconds steps to atrial ERP), sensed triples S1 = 600 milliseconds; S2 =ERP + 50 milliseconds, S3 =ERP + 50 milliseconds, decreasing in 10 millisecond steps to atrial ERP) and incremental pacing, decreasing in 10 millisecond intervals from 450 milliseconds to loss of 1:1 atrial capture.^13^ Each step in the induction protocol was carried out once before moving onto the subsequent step. If AF was not induced by this protocol the entire protocol was repeated for a second time. Sustained AF was defined as AF continuing for greater than 30 seconds. If AF resolved to an organized tachycardia, overdrive pacing was used to terminate the tachycardia. Sustained AF was terminated by electrical cardioversion if required.

### 2.4 Calculation of LA_ECS_

**Figure 1:**
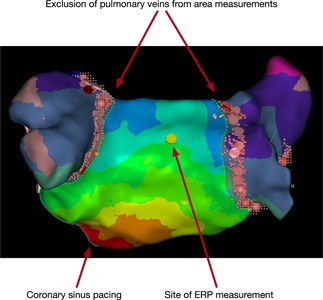
Estimation of post‐ablation LA body area and the typical site for ERP measurement. LA body area was calculated from Carto shells by subtracting the area of the isolated pulmonary veins (shaded), the left atrial appendage, and the mitral annulus from the total area of the LA shell. The yellow tag indicates the site at which ERP measurements and AF induction protocol were performed. The activation map was recorded during coronary sinus pacing. AF, atrial fibrillation; ERP, effective refractory period; LA, left atrial

Carto3 maps were analyzed offline and used to calculate left atrial body atrial area, total activation time, and global CV. Left atrial body area (*A*) was calculated by subtracting the area of the isolated pulmonary veins, the left atrial appendage, and the mitral annulus from the total area of the left atrial shell (Figure 1). Total activation time (*T*) was calculated by subtracting the earliest from the latest activation time point as annotated on the isochronal activation maps. Using $\sqrt{A}$ and T as the characteristic length and time, respectively, we defined the characteristic CV as

$$\mathrm{CV}=\frac{\sqrt{A}}{T}$$

Wavelength was defined as the shortest circuit that can sustain re‐entry and was calculated as

λ=CV×ERP

Left atrial effective conducting size (LA_ECS_, units = cm) was then calculated as

$${\mathrm{LA}}_{\text{ECS}}=\frac{A}{\lambda }$$

### 2.5 Atrial multiple wavelets model

Two dimensional (2D) and three dimensional (3D) computational modeling. For the 2D model, an 11 × 10 cm atrial tissue model was meshed using quadrilateral elements (1,100,000 elements, 1,102,101 nodes, average edge length ∆l= 0.1 mm). Electrical propagation was modeled using the mono‐domain equation, with isotropic conductivities of 0.3 S/m, giving CV= 77 cm/s. The cellular ionic current was modeled using the Luo‐Rudy cellular model, with ionic conductances modified to reproduce atrial cellular properties following Virag et al (*G*_Na_ = 16 mS/cm^2^, *G*_K_ = 0.423 mS/cm^2,^ and *G*_Si_ = 0.085 mS/cm^2^).^14^, ^15^ AF is sustained in this model by multiple wavelets undergoing functional re‐entry and wave break.^15^ The ionic conductance of I_K1_ was modified to reproduce the average ERP of the inducible persistent AF patients in this study (*G*_K1_ = 1.8 mS/cm^2^), to attain ERP = 231 milliseconds. AF was initiated by cross‐field stimulation. The numerical simulations were performed with CARPentry (https://carp.medunigraz.at/carputils/). To model the effects of pulmonary vein isolation, tissue at the left and right‐hand edges of the domain were set to be nonconductive (0.001 S/m). The effect of reducing left atrial body area by 10 to 60 cm^2^ was modeled by setting between 0.5 to 3.0 cm at each side of the domain to be nonconductive. To model the effects of changes in ERP (for example by the administration of sotalol), I_K1_ conductance was modified. Following Mitchell et al, we modeled ERP prolongation by sotalol in the range 290 to 330 milliseconds.^16^

3D simulations were performed on three left atrial anatomies generated from cardiac magnetic resonance sequences representing a range of left atrial body surface areas: 75, 103, and 140 cm^2^. The pulmonary veins were clipped (https://www.paraview.org) and meshes created using MMG tools software (http://www.mmgtools.org/). Endocardial atrial fiber direction information was mapped from an atlas geometry,^17^ and modeled with longitudinal conductivity 0.4 S/m and transverse conductivity 0.1 S/m (resultant CV = 0.77 m/s). As for the 2D simulations, sotalol administration was modeled by varying the ionic conductance of I_K1_ to produce ERP values in the range of 230 to 270 milliseconds. Ablation was modeled during arrhythmia simulations by setting ablation region conductivities as nonconductive (0.001 S/m). The following ablation patterns were applied: (a) pulmonary vein isolation alone via wide‐area encirclement of the pulmonary veins and (b) pulmonary vein isolation together with roof and inferior lines to model posterior wall box isolation. To vary the area of the isolated tissue the inferior line was applied at increasingly inferior locations on the posterior wall.

### 2.6 Statistical analysis

Data analysis were performed using SPSS statistics (IBM, Version 22) and Prism (GraphPad Software, Version 7). Continuous variables were expressed as a mean ± standard deviation. Comparison of means between groups was performed using the Mann‐Whitney *U* test for independent samples. Categorical variables were compared using *χ* ^2^ test. *P* < .05 was considered statistically significant.

## 3 RESULTS

### 3.1 Demographics and procedural details

Thirty‐nine patients (21 paroxysmal AF, 18 persistent AF) undergoing first‐time AF ablation were studied (Table 1). Mean AF history was 28 ± 37.4 months. Thirty patients were receiving rate‐controlling agents periprocedurally (β‐blockers n = 26; verapamil n = 3, digoxin n = 3) and 16 were taking antiarrhythmic medications (amiodarone n = 4; flecainide n = 10, others n = 2). Sustained AF was inducible in 14 (36%) patients overall (7 paroxysmal AF, 7 persistent AF).

**Table 1:** Summary of population demographics and measured and calculated study parameters according to AF category and AF vulnerability

		AF category			AF vulnerability		
Parameter		PAF	PsAF	*P*	Non‐inducible	Inducible	*P*
Age, y		56.2 ± 11.5	65.6 ± 15.1	.004	61.4 ± 13	59.1 ± 15.8	.817
Male sex, %		61.9	72.2	.337	62.5	78.6	.304
Paroxysmal AF, %		n/a	n/a	n/a	56	50	.718
Rate control, %		71.4	83.3	.379	76	78.6	.855
Rhythm control, %		52.4	27.8	.119	40	42.9	.862
LA body area, cm^2^		75.3 ± 11.6	98.6 ± 18	<.001	80.7 ± 16.4	95.5 ± 19.6	.0167
ERP, ms		259.5 ± 48.2	266.1 ± 67.4	.667	272.4 ± 56.7	245 ± 55.7	.118
CV, m/s		1.0 ± 0.2	1.0 ± 0.6	.140	1.1 ±;0.5	1.0 ± 0.2	.784
λ, cm		27 ± 7.8	27 ± 11.7	.707	28.4 ± 9.8	24.2 ± 7.9	.141
LA_ECS_, cm		3.2 ± 1.4	4.4 ± 2.0	.049	3.3 ± 1.7	4.4 ± 1.8	.035

Abbreviations: AF, atrial fibrillation; CV, conduction velocity; ERP, effective refractory period; LA, left atrium; LA_ECS_, left atrial effective conducting size; PAF, paroxysmal AF; PsAF, persistent AF; λ, wavelength.

### 3.2 Measured parameters and calculated parameters

Mean left atrial body area was 86.1 ± 18.8 cm^2^. Mean ERP (BCL = 600 milliseconds) was 262.6 ± 57.2 milliseconds. Mean left atrial total activation time was 97.1 ± 27.7 milliseconds. Calculated mean CV was 1.0 ± 0.4 m/s. Calculated mean LA_ECS_ was 3.7 ± 1.8 cm (Table 1).

#### 3.2.1 Category of AF

**Figure 2:**
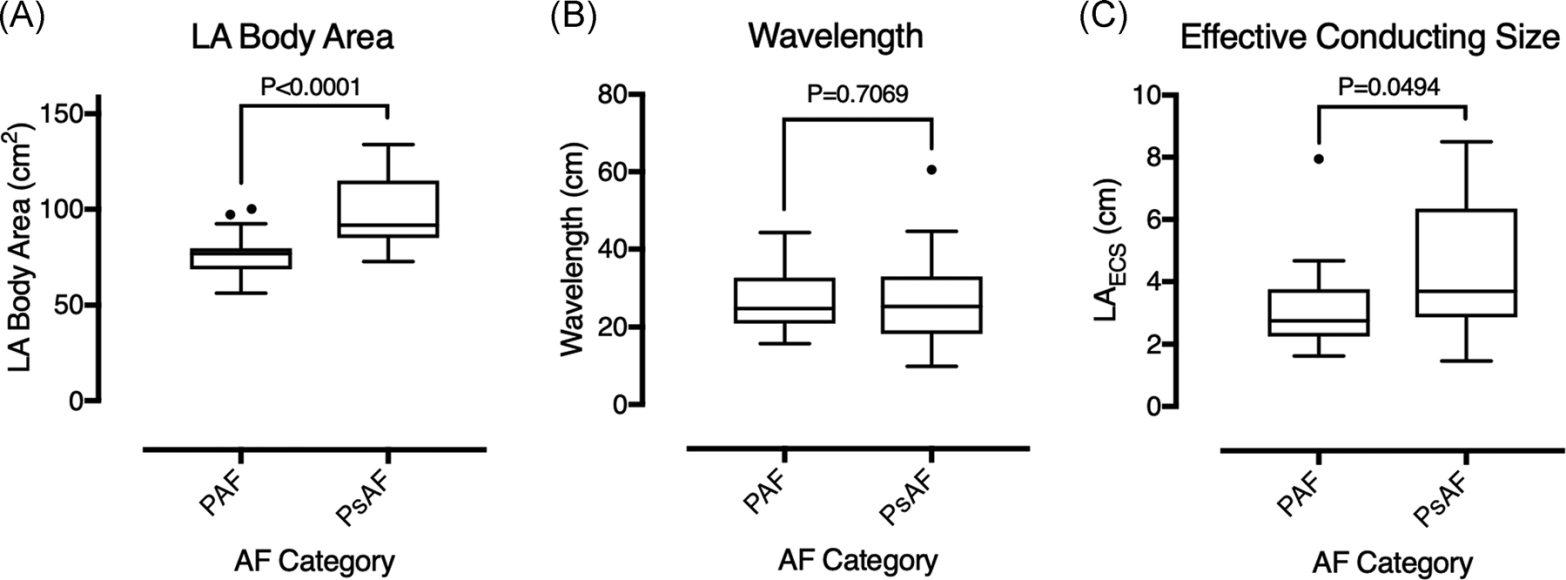
LA body area, calculated wavelength, and effective conducting size in patients with paroxysmal and persistent AF. A, LA body area was significantly greater in patients with persistent AF than paroxysmal AF. B, There was no significant difference in the calculated wavelength between patients with paroxysmal and persistent AF. C, Effective conducting size was significantly greater in patients with persistent than paroxysmal AF. AF, atrial fibrillation; LA, left atrium; LA_ECS_, LA effective conducting size; PAF, paroxysmal AF; PsAF, persistent AF

LA_ECS_ was significantly greater in patients with persistent AF than paroxysmal AF (4.4 ± 2.0 cm vs 3.2 ± 1.4 cm; *P* = .049; Figure 2) owing to a significantly greater left atrial body area in persistent AF patients (98.6 ± 18.0 cm^2^ vs 75.3 ± 11.6 cm^2^; *P* < .001). Left atrial total activation time was significantly longer in patients with persistent AF than paroxysmal AF (109 ± 30 milliseconds vs 87 ± 21 milliseconds; *P* = .005) but there were no significant relationships between ERP (266 ± 67 milliseconds vs 260 ± 48 milliseconds; *P* = .667), CV (1.0 ± 0.6 m/s vs 1.0 ±;0.2 m/s; *P* = .14) or wavelength (27 ± 11.7 cm vs 27 ± 7.8 cm; *P* = .707) and AF category.

#### 3.2.2 AF vulnerability

**Figure 3:**
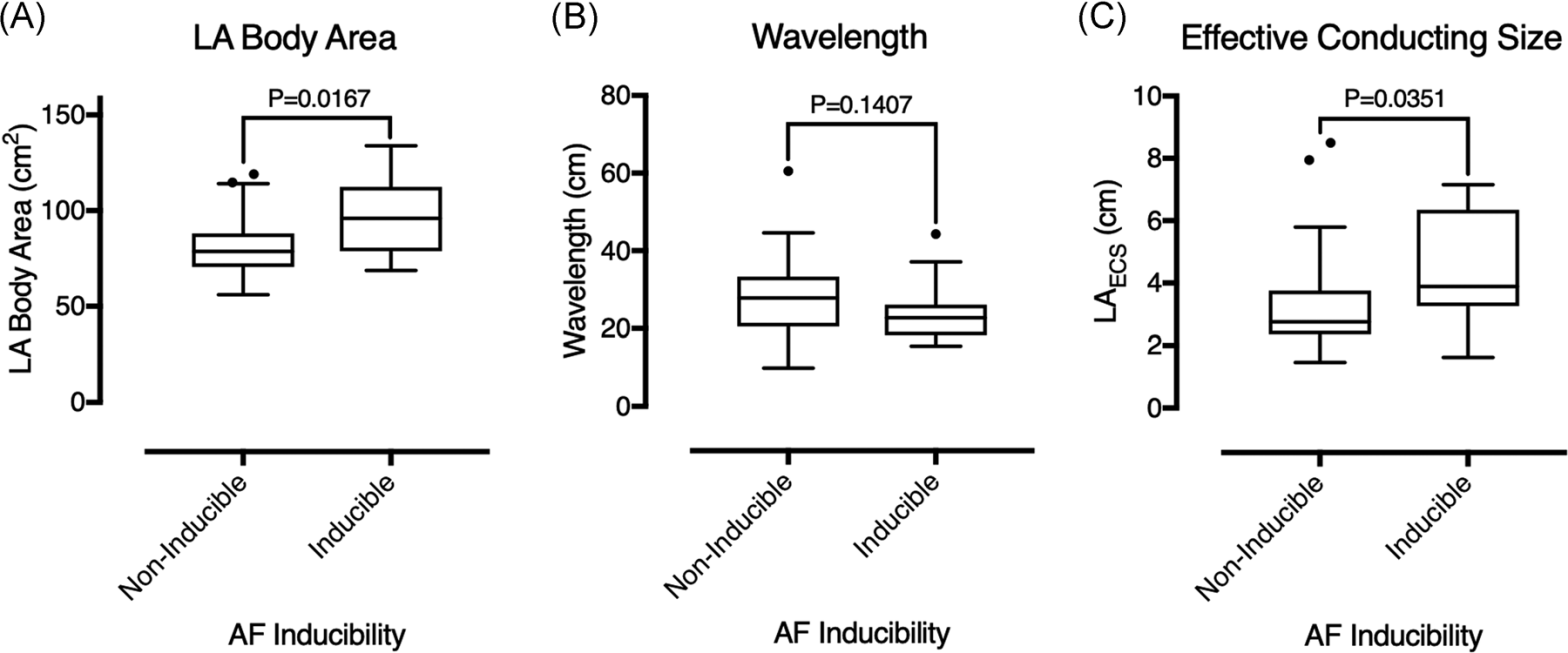
LA body area, calculated wavelength, and effective conducting size in patients with and without inducible AF. A, LA body area was significantly greater in patients with inducible vs. noninducible AF. B, There was no significant difference in wavelength between patients who were and were not AF inducible. C, Effective conducting size was significantly greater in patients with inducible vs noninducible AF. AF, atrial fibrillation; LA, left atrium; LA_ECS_, LA effective conducting size; PAF, paroxysmal AF; PsAF, persistent AF

Overall, LA_ECS_ was significantly greater in patients in whom AF was inducible than patients in whom AF was not inducible (4.4 ± 1.8 cm vs 3.3 ± 1.7 cm; *P* = .035; Figure 3). Left atrial body area was significantly greater in AF‐inducible patients than in noninducible patients (95.5 ± 19.6 cm^2^ vs 80.7 ± 16.4 cm^2^; *P* = .0167) but there were no significant relationships between ERP (245 ± 56 milliseconds vs 272 ± 56 milliseconds; *P* = .118), CV (1.0 ± 0.2 m/s vs 1.1 ±;0.5 m/s; *P* = .784) or wavelength (24.2 ± 7.9 cm vs 28.4 ± 9.8 cm; *P* = .141) and AF inducibility. There was no significant relationship between AF type and ability to induce AF (paroxysmal AF 33.3% inducible vs 67.7% noninducible; persistent AF 38.9% inducible vs 61.1% noninducible; *P* = .718).

#### 3.2.3 AF vulnerability according to AF type

**Figure 4:**
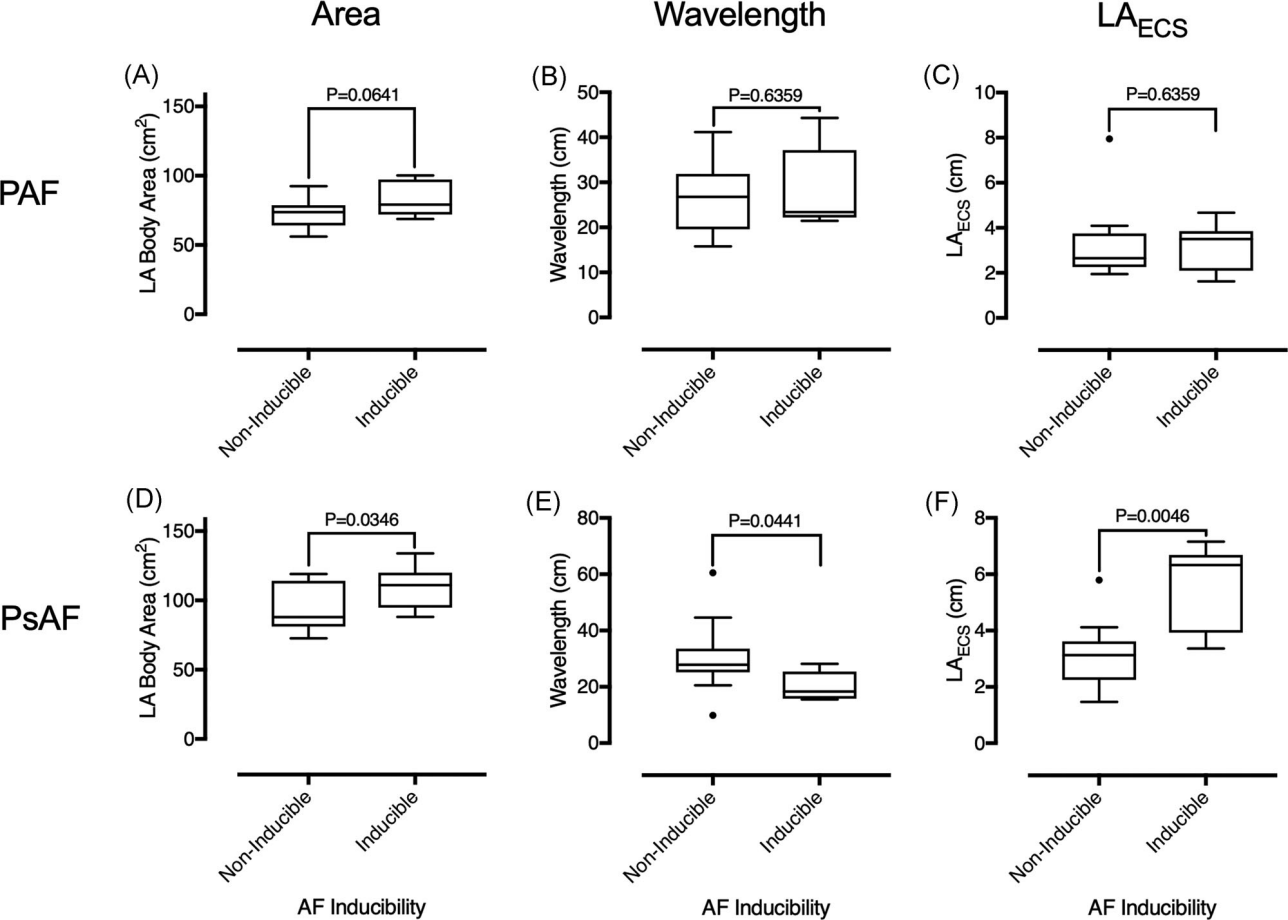
LA body area, calculated wavelength, and effective conducting size in patients with and without inducible AF, by AF class. There was no significant difference in LA body area (A), wavelength (B) or left atrial effective conducting size (C) between paroxysmal AF cases who were and were not AF inducible. LA body area (D) and left atrial effective conducting size (F) was significantly greater, and calculated wavelength (E) was significantly smaller in persistent AF cases who were AF inducible compared to those who were not AF inducible. AF, atrial fibrillation; LA, left atrium; LA_ECS_, LA effective conducting size; PAF, paroxysmal AF; PsAF, persistent AF

**Figure 5:**
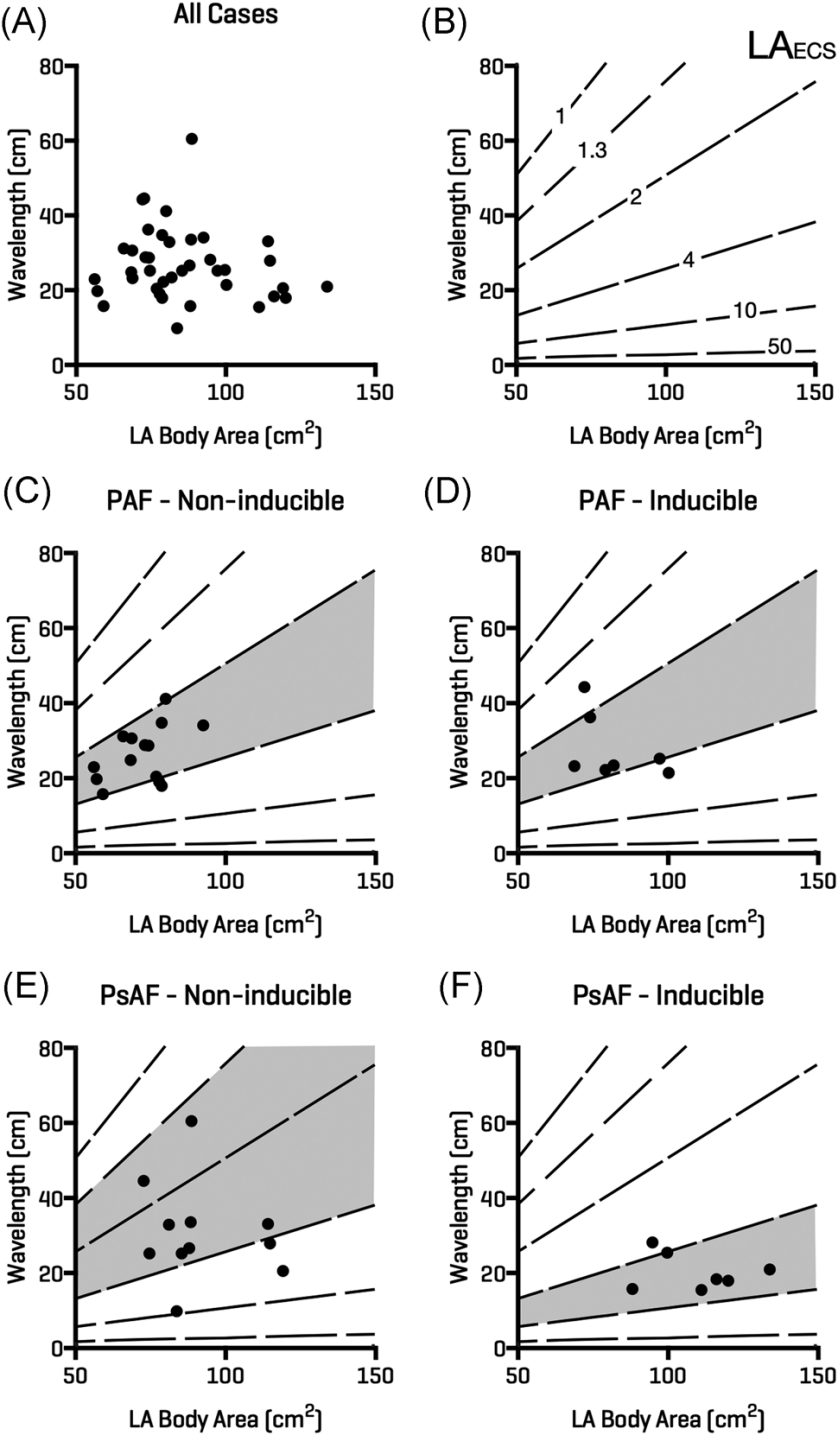
Relationship between induction of AF and LA body area, calculated wavelength and effective conducting size. A, LA body area is plotted on the *x*‐axis and calculated wavelength (see text) on the *y*‐axis for all patients in the study. B, Isolines represent LA_ECS_. Patients with paroxysmal AF who were both AF inducible (D) and AF noninducible (C) localized around the 2 to 4 cm LA_ECS_ region (shaded). Patients with persistent AF who were noninducible occupied a larger range of effective conducting size from 1.3 to 10 cm (E). Patients with persistent AF who were AF inducible largely fell within the >4 cm range for LA_ECS_. AF, atrial fibrillation; LA, left atrium; LA_ECS_ , LA effective conducting size; PAF, paroxysmal AF; PsAF, persistent AF

Left atrial body area was significantly greater (109.1 ± 15.9 cm^2^ vs 91.9 ± 16.4 cm^2^; *P* = .0346) and wavelength was significantly shorter (20.3 ± 4.8 cm vs 30.9 ± 13.1 cm; *P* = .0441) for persistent AF cases who were inducible compared to those who were noninducible (Figure 4). The significant relationship between LA_ECS_ and AF inducibility was driven by persistent AF (5.6 ± 1.4 cm vs 3.6 ±;2 cm; *P* = .0046) but not paroxysmal AF (3.2 ± 1.1 cm vs 2.1 ± 1.5 cm; *P* = .6359) cases. Wavelength is plotted against left atrial body area in Figure 5. Patients with paroxysmal AF who were both inducible and noninducible localized between the 2 to 4 cm LA_ECS_ isolines. In contrast, patients with persistent AF who were inducibly localized below the 4 cm isoline (ie LA_ECS_ > 4 cm).

#### 3.2.4 Simulated data

##### 2D simulations

**Figure 6:**
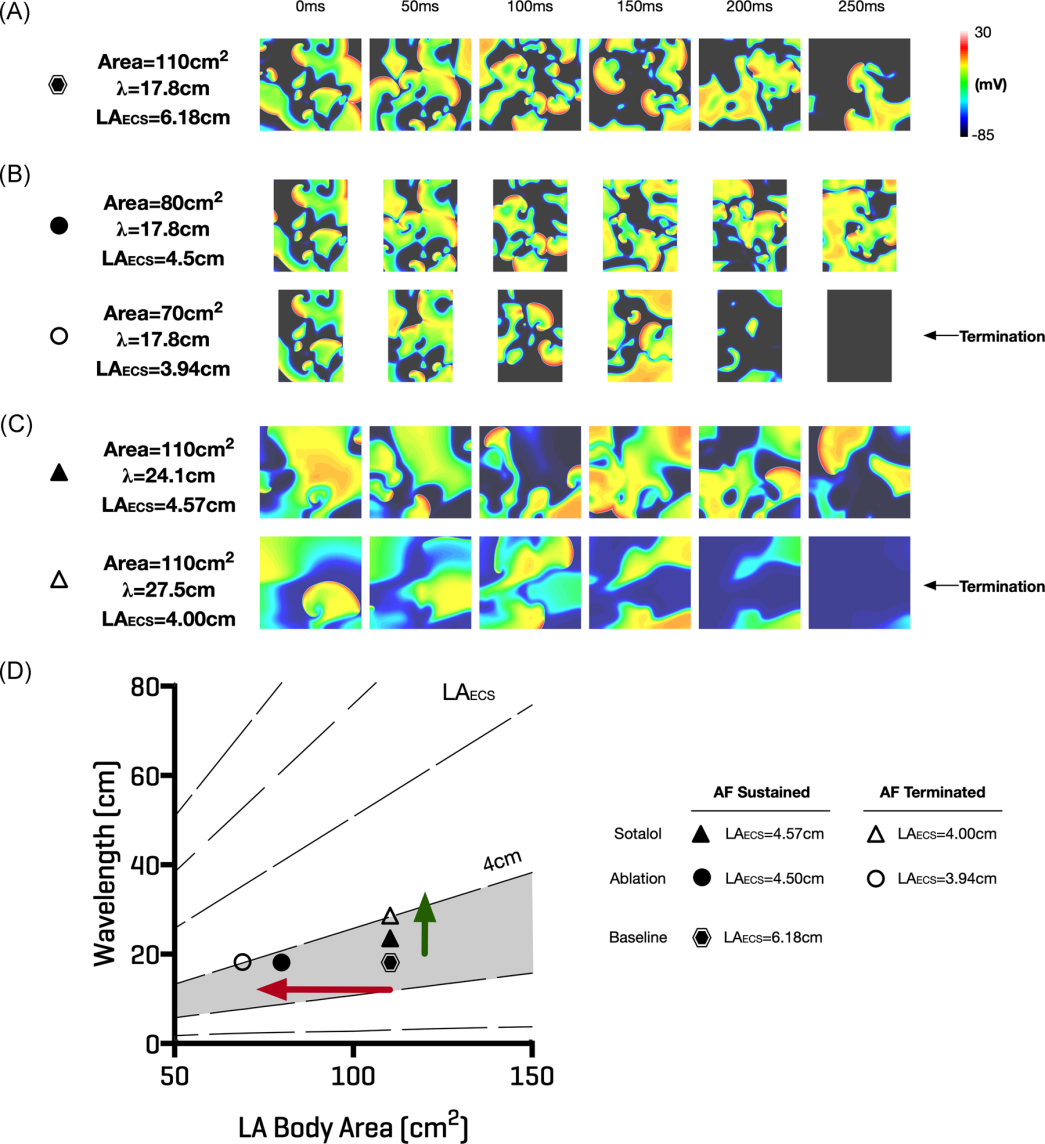
Effect of ablation and antiarrhythmic drugs on effective conducting size. Computational modeling confirmed that a critical LA_ECS_ > 4 cm was required for sustained multiple wavelet re‐entry. A, Re‐entry was sustained with LA_ECS_ = 6.18 cm (hexagon in “D”). B, Effect of reducing the atrial area to 80 cm^2^ (top row, continued AF, LA_ECS_ = 4.5 cm, closed circle in D) and 70 cm^2^ (bottom row, AF termination, LA_ECS_ = 3.94 cm, open circle in D). C, Effect of increasing sotalol doses on LA_ECS_. AF continued when LA_ECS_ > 4 cm (top row, closed triangle in D) but terminated with LA_ECS_ = 4.0 cm (bottom row, open triangle in D). D, Illustrates how ablation or antiarrhythmic drugs could be applied to alter LA_ECS_ and remove patients from the region of vulnerability (shaded grey). Ablation would reduce left atrial body area with a corresponding reduction in LA_ECS_ (red arrow). Conversely, antiarrhythmic drugs to prolong refractoriness could be applied to increase LA wavelength (green arrow) and decrease LA_ECS_. AF, atrial fibrillation; LA_ECS_ , LA effective conducting size

Baseline parameter sets were chosen to model multiple wavelet re‐entry with the ERP tuned to match the mean value for the inducible persistent AF patients. AF was sustained for this set‐up (Figure 6A) with a calculated LA_ECS_ = 6.18 cm (filled hexagon; Figure 6D). The effects of ablation and antiarrhythmic drugs on arrhythmia inducibility were tested by altering the conducting area of the domain and I_K1_ channel conductance, respectively. When atrial area was modified by ablation, a critical LA_ECS_ > 4 cm was required for maintenance of AF. AF was sustained for atrial areas of 80 cm^2^ (LA_ECS_ = 4.5 cm, filled circle; Figure 6D) or larger, whilst reducing the atrial area to 70 cm^2^ (LA_ECS_ = 3.9 cm; empty circle; Figure 6D) resulted in AF termination (Figure 6B). When modifications in ERP and CV were modeled by the application of sotalol, a critical LA_ECS_ > 4 cm was still required for maintenance of AF. AF was sustained at an ERP of 290 milliseconds (LA_ECS_ = 4.6; filled triangle; Figure 6D), but not at an ERP of 320 milliseconds (LA_ECS_ = 4 cm; empty triangle; Figure 6D) (Figure 6C). Modifying ERP through changes in G_K1_ conductance also modified CV, such that CV = 86 cm/s at ERP = 320 milliseconds and CV = 83 cm/s at ERP = 290 milliseconds.

##### 3D simulations

**Figure 7:**
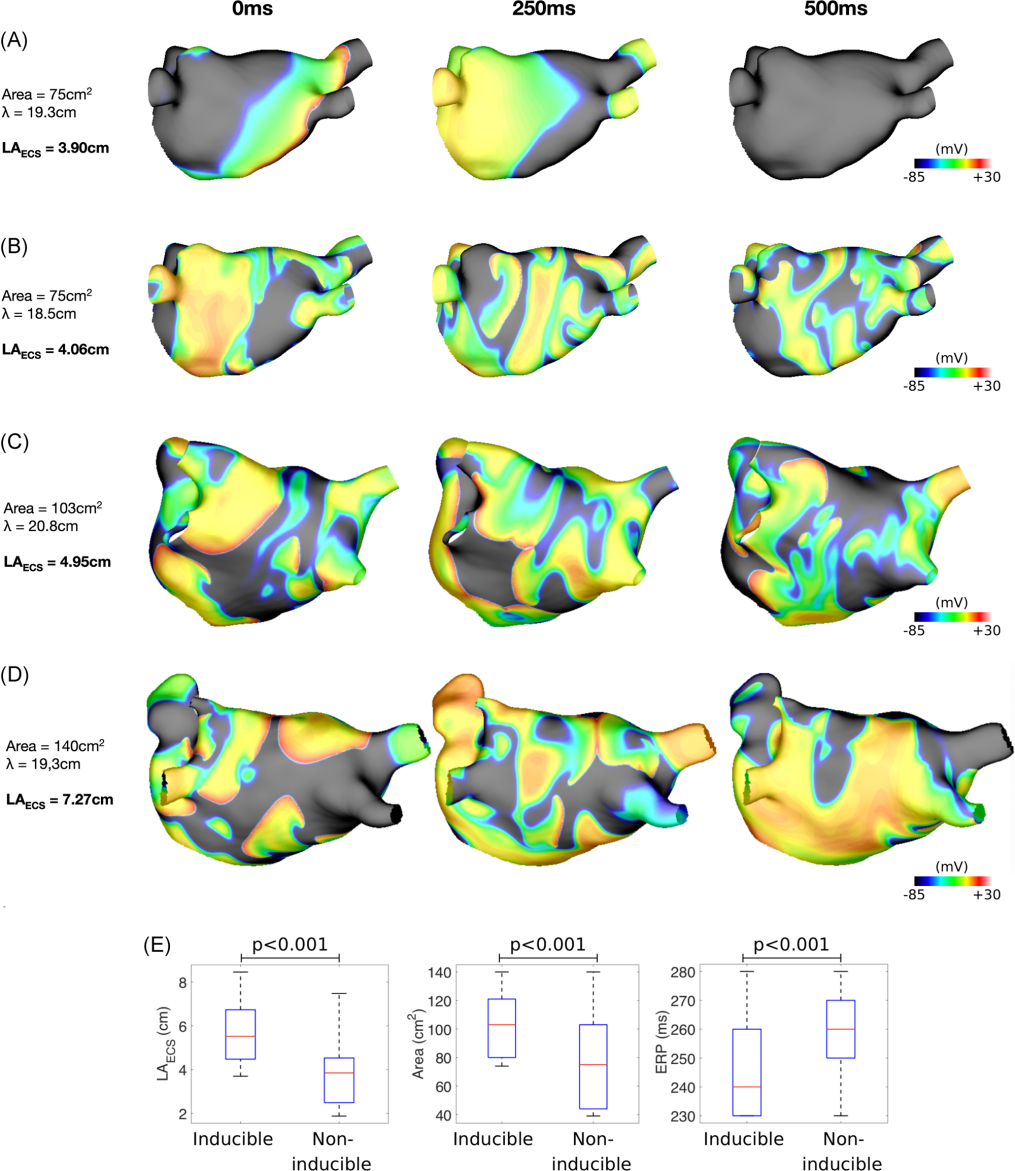
Effect of left atrial effective conducting size on arrhythmia inducibility in patient‐specific simulations. A, For the smallest anatomy (area 75 cm^2^), AF is noninducible for an ERP value of 250 milliseconds and LA_ECS_ = 3.90 cm. Isopotential plots are shown for time points at 250 milliseconds intervals. B, For the same anatomy as “A”, AF is inducible for an ERP value of 240 milliseconds and LA_ECS_ = 4.06 cm. C, For the medium anatomy (area 103 cm^2^), AF is inducible for a longer ERP (270 milliseconds) than for the smallest anatomy shown in “B”. D, For the largest anatomy (area 140 cm^2^), AF is inducible for all tested values of ERP. The example here is for an ERP of 250 milliseconds (as in “A”) and LA_ECS_ = 7.27 cm. E, LA_ECS_, left atrial body area and ERP are shown for inducible and noninducible simulations. Median LA_ECS_ is significantly lower for noninducible cases (3.85 cm vs 5.52 cm; *P* < .001), median left atrial body area is significantly lower for noninducible cases (75 cm^2^ vs 103 cm^2^; *P* < .001) and median ERP is significantly higher for noninducible cases (260 milliseconds vs 240 milliseconds; *P* < .001). AF, atrial fibrillation; ERP, effective refractory period; LA_ECS_ , LA effective conducting size

For the smallest geometry, ERP values of 250 to 270 milliseconds resulted in LA_ECS_ < 4 cm and for these simulations arrhythmias were not sustained (Figure 7A); whereas in simulations with an ERP of either 230 milliseconds (LA_ECS_ = 4.23 cm) or 240 milliseconds (LA_ECS_ = 4.06 cm; Figure 7B) AF was sustained. In comparison, for both the medium and large anatomies, ERPs between 230 and 270 milliseconds all resulted in LA_ECS_ > 4 cm and AF was correspondingly sustained in all cases (Figure 7C,D). Figure 7E shows LA_ECS_, ERP, and left atrial body area for inducible and noninducible cases. The median LA_ECS_ for noninducible cases is 3.85 cm, compared to 5.52 cm for inducible cases (*P* < .0001). The median ERP and area values are also significantly different between inducible and noninducible cases.

**Figure 8:**
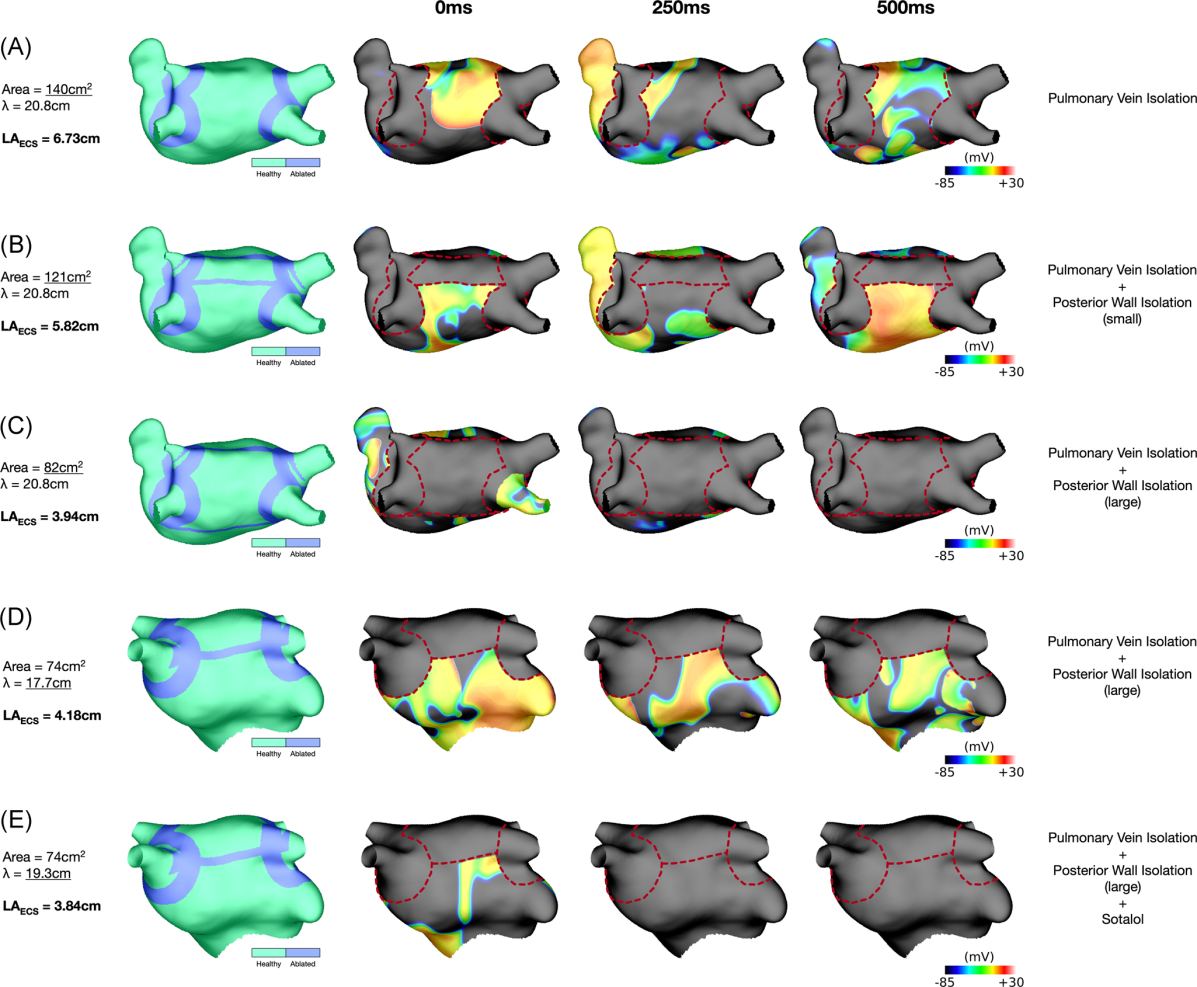
Left atrial effective size‐based arrhythmia therapy insights from patient‐specific simulations. A, AF is inducible post‐pulmonary vein isolation for the largest anatomy (area 140 cm^2^) with an ERP of 270 milliseconds (LA_ECS_ = 6.73 cm). Isopotential plots are shown for time points at 250 milliseconds intervals. B, AF is inducible after applying the smallest box isolation ablation to the largest anatomy (remaining left atrial body area, 121 cm^2^) with an ERP of 270 milliseconds (LA_ECS_ = 5.82 cm). C, AF is noninducible after applying the largest box isolation ablation to the largest anatomy (remaining left atrial body area, 82 cm^2^) with an ERP of 270 milliseconds (LA_ECS_ = 3.94 cm). Together “B” and “C” show that reducing the remaining left atrial body area is a viable therapeutic strategy to reduce LA_ECS_ and AF inducibility. D, AF is inducible after applying the largest box isolation ablation to the medium anatomy (remaining left atrial body area, 74 cm^2^) with an ERP of 230 milliseconds (LA_ECS_ = 4.18 cm). E, Modifying the ERP to 250 milliseconds for the case in “D” results in non‐inducibility (LA_ECS_ = 3.84 cm). Together “D” and “E” show that in certain cases combined the use of ablation and antiarrhythmic drugs may be needed to achieve the necessary reduction in LAECS and prevent AF inducibility. Dotted red lines indicate ablation lesion trajectories. “A”‐“C” (largest anatomy) are shown in posteroanterior orientation. D‐E, (medium anatomy) are shown in anteroposterior orientation with cranial tilt. AF, atrial fibrillation; ERP, effective refractory period; LA_ECS_, LA effective conducting size

In 76% of the simulations, AF sustained after pulmonary vein isolation (Figure 8A). Applying a box isolation lesion set further reduced LA_ECS_ and terminated AF in 67% of simulation set‐ups using the inferior line that resulted in the lowest LA_ECS_. Figure 8B shows an example in which AF was inducible with the smallest area of isolated tissue owing to a resultant atrial area of 121 cm^2^ and corresponding LA_ECS_ of 5.82 cm. Figure 8C shows the same anatomy, ERP and CV properties as Figure 8B. In this case, increasing the area of the isolated posterior box resulted in a LA_ECS_ of 3.94 cm, and termination of AF. In contrast, Figure 8D demonstrates a case in which AF is still inducible following the largest box isolation lesion set with an ERP of 230 milliseconds and LA_ECS_ remaining above 4 cm (LA_ECS_ = 4.18 cm). By additionally increasing ERP to 250 milliseconds, through modeling the effects of sotalol, LA_ECS_ is decreased less than 4 cm (LA_ECS_ = 3.84 cm) and AF terminates (Figure 8E).

## 4 DISCUSSION

In this study, we sought to design and test a clinically applicable technique to quantify atrial electrical substrate size determined using an atrial activation map and a single measurement of ERP. The main finding of the study is that LA_ECS_ is significantly greater in persistent, but not paroxysmal, AF patients who remain AF inducible after ablation. A critical LA_ECS_ > 4 cm is associated with inducibility of AF in both persistent AF patients and in 2‐ and 3‐dimensional simulation studies. Simulation studies also indicate that a combination of ablation and antiarrhythmic drugs may be required to reduce LA_ECS_ to <4 cm in certain patients. These findings support a future role for LA_ECS_ in guiding interventional, pharmacological or combined therapies for persistent AF.

### 4.1 Atrial size, electrical remodeling, and AF vulnerability

Left atrial dilatation is a well‐described risk factor for the development of AF and is associated with disease severity and outcome post ablation.^18^ These data demonstrate that left atrial body area is significantly associated with both AF severity (persistent vs paroxysmal AF) and AF vulnerability in persistent AF cases. The mechanisms by which atrial dilatation may increase AF vulnerability are incompletely understood. According to the multiple wavelet hypothesis, an increased atrial area would allow more re‐entrant circuits to exist^1^, ^19^ and thus increase susceptibility to AF. However, alterations in electrical properties could also be associated with atrial dilatation and contribute to the maintenance of AF.

Prior studies assessing the relationship between electrical remodeling and atrial dilatation have, however, yielded inconsistent results. Ravelli et al^20^ demonstrated shortened refractory periods in association with high atrial pressures in isolated rabbit atria. Conversely, Sparks et al^21^ demonstrated increased refractory periods in patients with chronic VVI pacing and increased atrial dimensions. Conduction slowing and block have been associated with acute dilatation in isolated rabbit atria^22^ and an association between reduced CV, atrial dilation, and arrhythmias has also been shown in human studies.^23^ In the present study, we found no significant relationship between left atrial size and ERP or total activation time in patients with either paroxysmal and persistent AF, although a nonsignificant trend toward shorter ERP and longer total activation time were noted in persistent patients with inducible vs noninducible AF. Therefore, there is a need to consider both atrial size and electrical conduction together (for example using LA_ECS_) in individual patients to predict response to treatments for arrhythmias.

### 4.2 The concept of Re‐entry, wavelength, and atrial substrate size

The concept of “atrial substrate size” was originally communicated by Moe in terms of a dimensionless quantity termed the fibrillation number.^19^ In a study by Hwang et al,^24^ fibrillation number, a function of wavelength and atrial diameter measured on 2D echo, was shown to be related to AF duration in computer simulations and AF induction cycle length in patients with AF undergoing ablation. Similarly, in an in vivo study in swine Lee et al^25^ demonstrated that the probability of sustained AF increased significantly with increasing tissue area and decreasing ERP. Wavelength, incorporating both refractory period and CV, has been shown to be a key determinant of re‐entry^5^ reliably predicting arrhythmia vulnerability in a canine study.^26^

Given the interaction between atrial size, refractoriness, CV, and AF vulnerability, we incorporated these parameters into a new metric termed LA_ECS_. This metric differs from the fibrillation number previously described^19^, ^24^ by using atrial surface area rather than a characteristic length constant such as atrial diameter which we believe is likely to better capture the complex geometrical remodeling present in AF patients.^27^ We extended the above findings to a population of AF patients by demonstrating that wavelength is significantly shorter and LA_ECS_ significantly greater in persistent AF patients with inducible vs noninducible AF (Figure 4F). LA_ECS_ also notably identified a population of patients with persistent AF who were not AF‐inducible after PVI and who, from an electrophysiological standpoint, appear to behave more like paroxysmal AF patients. Although this parameter ignores heterogeneity in refractoriness,^28^ quantifying such heterogeneity is time‐consuming and therefore not easily translated from research to clinical settings. Conversely, the data set required for calculation of LA_ECS_ in this study is easily obtainable within the timeframe of an ablation procedure and according to the present data is likely to represent a useful and clinically applicable metric for assessing AF vulnerability irrespective of the clinical AF categorization.

### 4.3 Clinical relevance

This study highlights the physiological difference between categories of AF and suggests a possible methodology by which arrhythmia management strategies could be individualized for patients post‐pulmonary vein isolation. In patients with LA_ECS_ < 4 cm, a trigger‐based ablation strategy may be most appropriate. In this study, LA_ECS_ < 4 cm incorporated the vast majority of patients with paroxysmal AF which is in line with success rates of pulmonary vein isolation in this population.^29^ Notably, however, even persistent AF patients with LA_ECS_ < 4 cm were noninducible after pulmonary vein isolation, suggesting that isolation of PV triggers could be successful in this subpopulation. Conversely, in patients with LA_ECS_ > 4 cm, additional substrate modification is likely to be required. In these patients, initial trigger‐dependent AF may have evolved to multiple wavelet/re‐entry based AF due to progressive structural and electrical remodeling.^30^ In recent years, additional catheter ablation strategies have been suggested in persistent AF patients^31^, ^32^, ^33^, however, the optimal treatment approach remains unclear.^34^ According to the present data targeted strategies to reduce LA_ECS_ (Figures 6D and 8) may improve the success of rhythm control strategies. In contemporary practice, a reduction in LA_ECS_ could be achieved by positioning the PV wide‐area encirclement lines more toward the center of the chamber, by isolating the LA posterior wall^35^ or by the selective use of antiarrhythmic drugs to prolong atrial refractoriness. Our modeling data highlights how in certain cases even a large posterior box isolation set does not reduce the LA_ECS_ sufficiently to terminate AF and this is only achieved through an additional increase in ERP. As such, knowledge of LA_ECS_ could be used to guide ablation during the initial ablation procedure or to guide the selection of additional ablation vs. antiarrhythmic drugs for the management of recurrent arrhythmia after initial ablation. Such management strategies require investigation in prospective randomized trials.

## 5 LIMITATIONS

This was a prospective simulation/clinical study to evaluate a new clinically applicable concept. Since there were a low number of recurrence events in the cohort, it was not possible to formally test the predictive power of LA_ECS_ in a statistical model including other clinical parameters associated with AF vulnerability and recurrence. ERP was measured at a single site in the left atrium which may not be fully reflective of the heterogeneity in refractoriness seen in patients with persistent AF. Some patients were maintained on antiarrhythmic drugs periprocedurally. While the effects of these in terms of CV and refractoriness will be accounted for in the LA_ECS_ concept, it is not possible to predict how discontinuation of medications post procedure may affect the vulnerability to AF in a particular patient from these data. Therefore, LA_ECS_ measured in the study may not reflect the true LA_ECS_ once antiarrhythmic drugs are either discontinued or resumed post procedure. This study focused on multiple wavelet mechanisms of AF only. It is possible that this hypothesis may hold for other forms of re‐entry in including micro‐entrant circuits and rotors however this was not explored in the modeling work carried out here. Similarly, we did not examine the potential roles of the right atrium in arrhythmogenesis in these patients.

## 6 CONCLUSION

LA_ECS_ was significantly associated with the ability to induce AF in patients post first‐time ablation for persistent, but not paroxysmal AF. These data support the mechanistic hypotheses of re‐entry in the population of persistent AF patients studied. The LA_ECS_ metric may be useful for predicting AF vulnerability and guiding arrhythmia management in such patients.

## CONFLICT OF INTERESTS

Professor O’Neill has received research support and honoraria from Biosense Webster and has received consultation fees from Medtronic, Biosense Webster, St. Jude/Abbott and Siemens. Dr. Niederer has received research support from St. Jude/Abbott, Boston Scientific, Roche, Pfizer and Siemens.

## AUTHOR CONTRIBUTIONS

Concept/design, data collection, data interpretation/analysis and critical revision of article: SW. Data collection, data interpretation/analysis, statistics, drafting of article: LO’N. Data collection, critical revision of article: CR. Data collection: JJ. Data collection: AM; Data collection: BR; Critical revision of article: RM; Critical revision of article: IS; Critical revision of article, statistics: JW. Data collection: MW. Critical revision of article: SN; Data collection: CS; Concept/design, data collection, critical revision of article, approval of article: MO’N.
